# Physiological and Molecular Responses of the Flag Leaf Under *L*-Phenylalanine Ammonia-Lyase Inhibition

**DOI:** 10.3390/cells14171368

**Published:** 2025-09-02

**Authors:** Katarzyna Hura, Michał Dziurka, Tomasz Hura, Magdalena Wójcik-Jagła, Andrzej Zieliński

**Affiliations:** 1 Department of Plant Breeding, Physiology and Seed Science, Faculty of Agriculture and Economics, Agricultural University, Podłużna 3, 30-239 Kraków, Poland; magdalena.wojcik-jagla@urk.edu.pl (M.W.-J.); andrzej.zielinski@urk.edu.pl (A.Z.); 2 Polish Academy of Sciences, The Franciszek Górski Institute of Plant Physiology, Niezapominajek 21, 30-239 Kraków, Poland; michal.dziurka@gmail.com (M.D.); t.hura@ifr-pan.edu.pl (T.H.)

**Keywords:** flag leaf, *L*-phenylalanine ammonia-lyase, PAL inhibitor, phenolics, photosynthetic activity, antioxidants

## Abstract

The increasing challenge posed by climate change necessitates a deeper understanding of the plant metabolic pathways that influence productivity under varying environmental conditions. In this context, investigating the impact of *L*-phenylalanine ammonia-lyase (PAL) inhibition on flag leaf physiology in winter triticale provides valuable insights into mechanisms that may affect crop productivity. This study investigated the physiological and molecular responses of the flag leaf following the application of *4*-hydroxybenzoic acid hydrazide (HBH), a PAL inhibitor. It was hypothesized that PAL inhibition would redirect carbon flow towards carbohydrate synthesis at the expense of phenolic production, accompanied by alterations in photosynthetic performance and antioxidant responses. HBH was detected in flag leaf tissues and caused a significant reduction in phenolic content, along with a marked increase in soluble carbohydrate levels. HBH treatment strongly activated the antioxidant system, as evidenced by elevated levels of non-enzymatic antioxidants and the increased activity of antioxidant enzymes. Changes were also observed in chlorophyll fluorescence parameters and in the accumulation of proteins associated with CO_2_ fixation and the photosynthetic apparatus function. These findings demonstrate that PAL inhibition can substantially affect the redox balance and photosynthetic activity of the flag leaf during a critical period of plant development and yield formation.

## 1. Introduction

Ongoing climate changes, including an increasing frequency of extreme weather events, represent a significant challenge for agriculture globally [1]. The main consequences are abiotic and biotic stresses, which, although not the direct focus of this study, affect plant physiology, leading to growth and development disturbances and consequently yield loss [2]. These stress conditions trigger complex physiological and biochemical responses in plants, including alterations in both primary and secondary metabolism. A key aspect of this response is the reallocation of carbon, which can influence both stress recovery and yield potential [3]. Therefore, strategies aimed at improving plant productivity by supporting their recovery after stress periods, such as biostimulants [4], genetic improvements [5], conservation tillage [6], precision irrigation [7], and soil management [8], are particularly important. Among such approaches is the application of specific metabolic regulators, such as inhibitors of *L*-phenylalanine ammonia-lyase (PAL), which may indirectly influence carbohydrate metabolism and thus yield formation [3].

As a PAL inhibitor, *4*-hydroxybenzoic acid hydrazide (HBH) limits the biosynthesis of phenolic compounds by decreasing PAL activity [9]. The level of phenolic compounds largely depends on carbohydrate availability, as carbohydrates serve as the main carbon source for their synthesis. Glucose, the primary product of photosynthesis, constitutes a key carbon source for numerous metabolic pathways, including phenolics biosynthesis [10]. The shikimate pathway depends on the availability of two metabolites derived from earlier stages of glucose metabolism: phosphoenolpyruvate (PEP) and erythrose-*4*-phosphate. These compounds are used to synthesize shikimic acid, which is subsequently converted into chorismic acid, which can be used directly for the biosynthesis of aromatic amino acids (phenylalanine, tyrosine, and tryptophan) [11]. In the context of the secondary metabolism of phenolic compounds, phenylalanine is especially important as a direct PAL substrate, initiating the phenylpropanoid pathway that leads to the synthesis of a wide range of phenolic compounds [12]. Thus, the level of phenolic compounds is tightly linked to sugar availability and the activity of the shikimate pathway, which links the primary and secondary phenolic metabolism. However, to fully exploit the potential of PAL inhibitors in agriculture, it is necessary to understand their effects on plant physiology under optimal, non-stress conditions.

In cereals, the flag leaf plays a pivotal role as the primary source of photosynthesis products [13]. Due to its position and well-developed photosynthetic apparatus, it supplies the necessary carbohydrates to support organs that are strong carbohydrate sinks, such as developing spikes and grains [14]. The photosynthetic efficiency of the flag leaf directly impacts grain yield quality and quantity, as it determines the allocation of assimilates to the grains. Moreover, it remains photosynthetically active longer than the lower leaves of cereals, especially under optimal conditions, and is less prone to senescence [15]. Therefore, maintaining its photosynthetic activity for as long as possible is the key to enhancing cereal productivity. Given this central role, the flag leaf is often used as a model organ in physiological and biochemical studies assessing the effects of environmental stresses or growth regulators in cereals [16].

Triticale (×*Triticosecale* Wittmack) is a man-made, self-pollinating cereal crop species developed by crossing wheat (*Triticum* spp.) and rye (*Secale cereale*) [17]. Winter triticale plays a significant role as a feed grain, particularly in the diets of pigs and poultry. Climate change discussions typically prominently feature human food security, with the equally vital issue of feed availability, essential for maintaining stable livestock production, often receiving less attention [18,19].

Thus, the aim of this study was to determine the effect of PAL inhibition on the physiological and molecular condition of the flag leaf of winter triticale under optimal growth conditions. This study includes measurements of HBH content and the enzymatic activities of *L*-phenylalanine ammonia-lyase (PAL) and *L*-tyrosine ammonia-lyase (TAL), along with the quantification of soluble phenolic compounds and carbohydrates. Gas exchange, photosynthetic apparatus activity, chlorophyll contents, flag leaf biomass, and grain yield were also evaluated. At the molecular level, the accumulation of proteins involved in photosynthetic CO_2_ fixation (Rubisco activase—RCA, Rubisco large subunit—RbcL) and photosynthetic apparatus activity (photosystem II D_1_ protein—PsbA) were analyzed. Antioxidant activity was assessed by estimating the level of nonenzymatic antioxidants and the activity of enzymatic antioxidants. We hypothesized that under non-stress conditions, PAL inhibition would induce the redistribution of carbon towards soluble carbohydrate synthesis at the expense of phenolic compounds, accompanied by changes in the photosynthetic and antioxidant activity of flag leaves, ultimately affecting the flag leaf condition and final grain yield.

## 2. Materials and Methods

### 2.1. Plant Material and Growth Conditions

The experiments were conducted on the long-stemmed winter triticale cultivar ‘Moderato’, characterized by an intensive use of carbohydrates for phenolic compound biosynthesis [20]. Seeds were sourced from Danko Plant Breeders Ltd., Choryń, Poland. Seeds of triticale were sown into 3.7 L plastic containers, each filled with a standardized substrate consisting of a soil-to-sand mixture in a 1:3 (*v*/*v*) ratio. At the two-leaf developmental stage, seedlings were subjected to vernalization for 8 weeks in a controlled-environment chamber at 4 ± 1 °C. During vernalization, plants were exposed to a 10 h light/14 h dark photoperiod, with a photosynthetic photon flux density (PPFD) of 150 μmol m^−2^ s^−1^.

Following vernalization, plants at the four-leaf stage were relocated to a greenhouse chamber. Day/night temperatures were maintained at 26–28/18 ± 2 °C, respectively, with a relative humidity of approximately 40%. Light was supplied using high-pressure sodium lamps (400 W; Philips SON-T AGRO, Brussels, Belgium), delivering a PPFD of 180–200 μmol m^−2^ s^−1^ at canopy level. The water content in pots was maintained at 75%, corresponding to a soil water potential of −0.011 MPa, determined via a psychrometer HR 33 T (WESCOR, Inc., Logan, UT, USA). The soil water content was controlled daily between 9.00 a.m. and 10.00 a.m. using a gravimetric method (weight-based), taking into account plant weight. Throughout the growth period, plants received weekly irrigation with a complete Hoagland nutrient solution [21].

### 2.2. PAL Inhibition

The *L*-phenylalanine ammonia-lyase (PAL) inhibitor, *4*-hydroxybenzoic acid hydrazide (HBH), was applied at a concentration of 10^−3^ M. The HBH concentration (10^−3^ M) was chosen according to a previous study on wheat responses under biotic stress conditions [9]. Initial assays verified the suitability and safety of the applied HBH concentration for triticale flag leaves. The treatment was carried out daily in the morning over 12 consecutive days during the heading stage. The HBH solution was gently brushed onto the adaxial surface of fully expanded flag leaves using a soft brush to ensure a uniform distribution and minimize mechanical damage. Control plants were treated in the same manner with distilled water. All plants were maintained under identical environmental conditions throughout the treatment period.

### 2.3. Measurements

Leaf samples were collected 24 h after the completion of inhibitor treatment. Flag leaves from both control and inhibitor-treated plants were washed once with a 10% ethanol solution, followed by a rinse with distilled water to remove any residual inhibitor from the leaf surface. Samples were then immediately frozen in liquid nitrogen, lyophilized (Freeze Dry System/Freezone^®^ 4.5, LABCONCO Kansas City, MO, USA), and ground into a fine powder (MM400, Retsch, Haan, Germany). The powdered material was subsequently used for biochemical analyses.

#### 2.3.1. Leaf Water Potential (Ψ_W_) and Leaf Osmotic Potential (Ψ_O_)

Water potential (Ψ_W_) and osmotic potential (Ψ_O_) were determined using a psychrometer HR 33T (WESCOR, Inc., Logan, UT, USA) equipped with C-52-type leaf chambers (WESCOR) and a digital Metex M-3640D meter. For water potential measurements, fresh leaf disks (5 mm diameter), excised from the central part of the leaf, were placed in the chambers and allowed to equilibrate for 45 min to saturate the chamber atmosphere with water vapor [22]. The measurements were taken in 7 replicates.

Osmotic potential was measured with use of paper disks (5 mm diameter) saturated with cell sap extracted from leaves using a medical syringe. These samples were similarly equilibrated in the chambers for 30 min prior to measurement. Both potentials were assessed by the dew point method [20]. The measurements were taken in 7 replicates.

#### 2.3.2. Hydroxybenzoic Acid Hydrazide Levels

To assess the concentration of HBH in plant tissues, approximately 50 mg of dry sample was subjected to two-step extraction using 1 mL of a methanol/water/concentrated ammonia mixture (MeOH:H_2_O:NH_4_OH; 20:70:10, *v*/*v*/*v*). The samples were vortexed and shaken for 10 min at 30 Hz. After extraction, centrifugation was performed for 5 min at 22,000× *g* and 10 °C using a Universal 32R centrifuge (Hettich, Tuttlingen, Germany). The resulting supernatants were pooled and concentrated under a nitrogen stream using a TurboVap LV evaporator (Capillary, MA, USA). The dry residue was dissolved in 1.2 mL of 0.1 M ammonium hydroxide (NH_4_OH), centrifuged again, and the clear supernatant was once more dried under nitrogen. The final dried extract was reconstituted in 150 µL of buffer composed of 65% acetonitrile in 100 mM ammonium acetate (NH_4_CH_3_COO, *v*/*v*). After the final centrifugation, samples were analyzed via ultra-high-performance liquid chromatography (UHPLC) on an Agilent Infinity 1260 system (Agilent, Waldbronn, Germany), coupled with a triple quadrupole mass spectrometer (Agilent 6410, Palo Alto, CA, USA) using electrospray ionization (ESI). Chromatographic separation was conducted on an ACE HILIC-N column (5 μm, 2.1 × 100 mm; ACE, Aberdeen, UK) using a linear gradient elution with phase A (H_2_O containing 10% 100 mM ammonium acetate, pH 9) and phase B (acetonitrile with 100 mM ammonium acetate, pH 9). The gradient was altered from 100% to 60% phase B over 1.5 min, maintained for 0.5 min, and then returned to initial conditions over 0.2 min. The flow rate was 0.5 mL/min and the column temperature was set at 40 °C. The mobile phase was supplemented with 5 μM medronic acid to improve peak shape and ionization efficiency. Detection was carried out using dynamic multiple reaction monitoring (MRM). The protonated precursor ion ([M^+^H]^+^) at *m*/*z* 153.1 was monitored, along with two fragment ions at *m*/*z* 95.1 (collision energy: 11 V) and *m*/*z* 81.1 (collision energy: 35 V), using a fragmentor voltage of 84 V. The ion source was operated under the following conditions: capillary voltage, 4 kV; drying gas temperature, 350 °C; flow rate, 12 L/min; and nebulizer pressure, 35 psi. Quantification was achieved by external calibration with an analytical-grade HBH standard. All solvents and reagents used were obtained from Sigma-Aldrich (Poznań, Poland). The measurements were taken in 7 replicates.

#### 2.3.3. Soluble Phenolics (SPh)

Approximately 5.0 mg of freeze-dried plant material was used for the quantification of soluble phenolics (SPh). Soluble phenolic compounds were extracted using 100 µL of 80% (*v*/*v*) ethanol. The total SPh content was assessed using the Folin–Ciocalteu method [23]. The reaction mixture consisted of 50 µL of the ethanolic extract, 950 µL of distilled water, 500 µL of 25% (*w*/*v*) sodium carbonate, and 125 µL of Folin–Ciocalteu reagent (pre-diluted 1:1 with distilled water). Quantification was performed using a standard curve prepared with chlorogenic acid, and absorbance was measured at 760 nm using a UV-Vis spectrophotometer (Ultrospec 2100 Pro, Amersham Biosciences, Cambridge, UK) [24,25]. The measurements were taken in 7 replicates.

#### 2.3.4. Activity of *L*-Phenylalanine Ammonia-Lyase (PAL) and *L*-Tyrosine Ammonia-Lyase (TAL)

The activities of *L*-phenylalanine ammonia-lyase (PAL) and *L*-tyrosine ammonia-lyase (TAL) were determined using a modified protocol based on the method described by Peltonen and Karjalainen [26]. Fresh plant material (approximately 100 mg) was homogenized in an extraction buffer composed of 50 mM Tris–HCl (pH 8.5), 14.4 mM *2*-mercaptoethanol, and 5% (*w*/*v*) polyvinylpyrrolidone (PVP) under cooled conditions (4 °C). For enzymatic assays, the reaction mixture consisted of 2.5 mL of a 0.2% solution of either *L*-phenylalanine (for PAL activity) or *L*-tyrosine (for TAL activity) in 50 mM Tris–HCl buffer (pH 8.5), and 0.5 mL of the enzyme extract (supernatant). The mixtures were incubated for 24 h at 38 °C. After incubation, absorbance was measured using a spectrophotometer (Ultrospec 2100 Pro, Amersham Biosciences, Cambridge, UK) at 290 nm and 310 nm for PAL and TAL activities, respectively. Enzymatic activity was expressed as the amount of cinnamic acid (for PAL) or *p*-coumaric acid (for TAL) formed per hour per milligram of protein (nmol h^−1^ mg^−1^ protein). The protein concentration in the extracts was determined according to the method of Bradford [27]. The measurements were taken in 7 replicates.

#### 2.3.5. Photosynthetic Activity

Gas exchange parameters were measured on fully expanded leaves under greenhouse conditions at an ambient temperature of 25  ±  2 °C. An open gas exchange system equipped with an infrared CO_2_ analyzer (CI-301, CID Inc., Vancouver, WA, USA) and a leaf chamber was used for the assessments. The net photosynthetic rate (P_N_), transpiration rate (E), stomatal conductance (*g*_S_) and intercellular concentration of CO_2_ (*C_i_*) were recorded between 11:00 and 13:00 h. During the measurements, the air flow rate was maintained at 200 mL min^−1^, and the leaves were exposed to a saturating photosynthetic photon flux density (PPFD) of 1100 μmol m^−2^ s^−1^ provided by a dedicated LED light source (CI-301LA, CID, Camas, WA, USA). The CO_2_ concentration within the chamber was kept constant at 400 ppm using a CO_2_ control module (CI-301AD, CID, Camas, WA, USA). The stomatal limitation value (L_S_) was then calculated using the following formula according to Berry and Downton: L_S_ = 1 − C*_i_*/C*_a_* [28]. The apparent carboxylation efficiency (A_m_) was calculated as follows: A_m_ = P_N_/C*_i_* [29]. The instantaneous water use efficiency index (WUE*_inst_*_._) represents the P_N_/E ratio, while the intrinsic WUE*_intr_*_._ represents the P_N_/*g*_S_ ratio [30]. The measurements were taken in 10 replicates.

#### 2.3.6. Chlorophyll Fluorescence Measurements

Chlorophyll fluorescence parameters were assessed using an FMS 2 pulse-modulated fluorometer (Hansatech Instruments, Kings Lynn, UK). Prior to the measurements, leaves were dark-adapted for 20 min. F_v_/F_m_ (quantum yield of PSII) was calculated according to van Kooten and Snel [31]. The initial fluorescence (F_o_) was recorded under weak, modulated red light (600 nm; <0.1 μmol m^−2^ s^−1^), which did not trigger a significant variable fluorescence response. The maximum fluorescence in the dark-adapted state (F_m_) was measured using a 0.7-s saturating light pulse (8000 μmol m^−2^ s^−1^). Variable fluorescence (F_v_) was then calculated as the difference between F_m_ and F_o_. Subsequently, the leaves were exposed to continuous white actinic light at 400 μmol m^−2^ s^−1^, and after seven minutes of illumination, the steady-state fluorescence (F_s_’) was recorded, followed by a second saturating pulse to determine the maximum fluorescence under light-adapted conditions (F_m_’). The photochemical quenching coefficient q_P_ = (F_m_’ − F_s_)/(F_m_’ − F_o_’), the efficiency of excitation transfer to open PSII centers F_v_’/F_m_’ = (F_m_’ − F_o_’)/F_m_’, the PSII quantum efficiency Φ_PSII_ = (F_m_’ − F_s_)/F_m_’ and the electron transport rate ETR = PAR × 0.5 × Φ_PSII_ × 0.84 were calculated as per Genty et al. [32]. Non-photochemical quenching, q_N_ = 1 − (F_m_’ − F_o_’)/(F_m_ − F_o_), was calculated according to van Kooten and Snel [31]. The measurements were taken in 7 replicates.

#### 2.3.7. Chlorophyll Content

Chlorophyll content was assessed using a handheld chlorophyll meter (CL-01, Hansatech Instruments Ltd., Kings Lynn, UK). Measurements were taken from the middle part of fully expanded leaves and taken in 7 replicates.

#### 2.3.8. Soluble Carbohydrate Content

The concentration of total soluble carbohydrates was assessed using the anthrone method [30]. Leaf tissue extracts were reacted with anthrone reagent prepared in concentrated sulfuric acid, and were subsequently incubated at 90 °C for 15 min. The resulting colorimetric reaction was quantified by measuring the absorbance at 620 nm using a spectrophotometer (Ultrospec 2100 Pro, Amersham Biosciences, Cambridge, UK). A standard calibration curve was constructed using glucose solutions. The measurements were taken in 7 replicates.

#### 2.3.9. Western Blot Analysis

The analysis involved proteins of the photosynthetic apparatus (PsbA, D_1_ protein of PSII, C-terminal − Agrisera AS05 084) and proteins responsible for photosynthetic fixation of CO_2_ (RA, Rubisco activase − Agrisera AS10 700; RbcL, Rubisco large subunit, form I and form II − Agrisera AS03 037). Proteins were precipitated using 10% (*w*/*v*) trichloroacetic acid (TCA) containing 0.07% (*v*/*v*) *β*-mercaptoethanol dissolved in acetone. Approximately 5 mg of the lyophilized proteins was resuspended in 280 μL of loading buffer (4% SDS, 12% glycerol, 2% *β*-mercaptoethanol, 0.01% bromophenol blue in 50 mM Tris-HCl, pH 6.8), boiled for 2 min, and then centrifuged at 12,000× *g* for 10 min. Subsequently, 30 μL of the supernatant was loaded onto 12% polyacrylamide separating gel with 5% stacking gel. SDS-PAGE electrophoresis was performed using the Mini-Protean Tetra Cell system (Bio-Rad, Hercules, CA, USA) at 195 V for 45 min for higher-molecular-weight proteins (RA, RbcL) and at 165 V for 55 min for PsbA. Following electrophoresis, proteins were transferred onto PVDF membranes (0.2 μm pore size; Bio-Rad) using a Trans-Blot SD semi-dry transfer apparatus. Transfer was conducted with a stepwise voltage protocol (10 V for 15 min, 13 V for 15 min, 15 V for 15 min) in Tris-glycine buffer (pH 9.0). Membranes were blocked overnight in 2% (*w*/*v*) non-fat milk in TBS, then incubated for 1 h with the primary antibody (goat anti-rabbit IgG, Agrisera AS09 607). After washing with TBS, membranes were exposed to an alkaline phosphatase-conjugated secondary antibody for 1 h. Protein–antibody complexes were detected using a BCIP/NBT substrate kit (Sigma-Aldrich). After staining, membranes were imaged using the MicroDOC gel documentation system (Cleaver Scientific Ltd., Warwickshire, UK) [33] and the band density was analyzed using Image Studio Litever 5.2 software (LI-COR, Lincoln, NE, USA) and normalized to the densest band (treated as 100%).

#### 2.3.10. Analysis of Antioxidant Capacity

To estimate the total antioxidant capacity, fresh plant material was lyophilized using an LGA05 freeze dryer (MLW, Leipzig, Germany, upgraded by JWE, Warszawa, Poland) and ground in an MM 400 mill (Retch, Haan, Germany) using zirconia oxide beads. Approximately 10 mg of the samples was extracted for 5 min at 1.5 Hz and 2–8 °C in 1 mL of cooled 50 mM phosphate–potassium buffer (pH 7) containing 0.1 mM EDTA (PK-EDTA). Samples were then centrifuged for 5 min at 22,000× *g* and 10 °C using a Universal 32R centrifuge (Hettich, Tuttlingen, Germany). The supernatant (enzyme extract, hydrophilic fraction) was collected and aliquoted to estimate soluble proteins, various total enzyme activities, and unspecific antioxidant activity using the CUPRAC (cupric ion reducing antioxidant capacity) method. The remaining pellet was re-extracted with 0.5 mL of methanol (lipophilic fraction), followed by two additional extractions with 0.75 mL each time, shaking at 15 Hz for 5 min, and centrifuging at 22,000× *g* and 10 °C. The methanolic supernatant was combined and further analyzed using CUPRAC. The remaining pellet (insoluble fraction) was used to measure the total antioxidant capacity of insoluble and matrix-bound matter using the QUENCHER-CUPRAC (quick, easy, new, cheap, and reproducible CUPRAC) method.

All analyses were conducted spectrophotometrically in a 96-well plate format on a Synergy 2 instrument (Biotek, Winooski, VT, USA) at 25 °C. UV-transparent 96-well plates (Greiner, Kremsmünster, Austria) were utilized, and all plastic utensils were low-retention and biocompatible (Mettler-Toledo) [34]. All reagents were supplied by Sigma-Aldrich.

##### Superoxide Dismutase (SOD, EC 1.15.1.1)

SOD activity was determined using the cytochrome reduction inhibition method [35]. Changes in the absorbance of cytochrome (0.025 mM), reduced by the peroxides generated by the xanthine/xanthine-oxidase system (1 mM/0.003 U) after the addition of the enzyme extract, were monitored at 550 nm [34] in PK-EDTA buffer, with the method scaled down to a 96-well plate format. The measurements were taken in 5 replicates.

##### Total Soluble Peroxidase (POX, EC 1.11.1.x)

POX activity was measured according to the method of Lück [36]. The increase in absorbance of oxidized *p*-phenylenediamine (0.25 mM) after the addition of enzyme extract and H_2_O_2_ (0.5 mM) was monitored at 460 nm [34] in PK-EDTA buffer, scaled down to a 96-well plate format. The measurements were taken in 5 replicates.

##### Catalase (CAT, EC 1.11.1.6)

CAT activity was measured according to Aebi [37] by monitoring the decrease in absorbance of H_2_O_2_ (25 mM) after the addition of enzyme extract at 240 nm [34] in PK-EDTA buffer, scaling-down to a 96-well plate format. The measurements were taken in 5 replicates.

##### Glutathione Peroxidase (GPx, EC 1.11.1.9)

GPx activity was measured according to Wendel [38], with the method scaled down to a 96-well plate format. The oxidation of glutathione (GSH, 2 mM) to glutathione (GSSG) was catalyzed by GPx and coupled to the recycling of GSSG back to GSH using glutathione reductase (GR, 0.5 U/mL) and NADPH (0.25 mM). The decrease in NADPH absorbance during its oxidation to NADP+ was monitored at 340 nm. The reaction was carried out at pH 8.0 (50 mM Tris HCl with 0.5 mM EDTA) and initiated with organic peroxide *tert*-butyl hydroperoxide (*t*-Bu-OOH, 0.3 mM). The spontaneous reaction between *t*-Bu-OOH and GSH is minimal and is not metabolized by catalase, making the reaction specific to selenium-containing GPx. The measurements were taken in 5 replicates.

##### Glutathione Reductase (GR, EC 1.6.4.2)

GR activity was determined according to Smith et al. [39] with a scale-down to a 96-well plate format. GR specifically recycles oxidized glutathione (GSSG, 1 mM) back to its reduced form (GSH) utilizing NADPH (0.1 mM). The *5,5′*-dithiobis(*2*-nitrobenzoic acid) (DTNB; 0.75 mM) reacts with GSH, resulting in the formation of *5*-thio(*2*-nitrobenzoic acid) TNB. The increase in absorbance at 412 nm was monitored. The reaction was conducted at pH 7.8 (200 mM Tris HCl with 0.5 mM EDTA) and the measurements were taken in 5 replicates.

##### *L*-Ascorbate Peroxidase (AsPOX, EC 1.11.1.11)

AsPOX activity was assayed following the method of Nakano and Asada [40], with the assay scaled down to a 96-well plate format. The oxidation of ascorbic acid (0.25 mM) was monitored at 290 nm in 200 mM Tris HCl buffer with 0.5 mM EDTA after the addition of 0.5 mM H_2_O_2_. The measurements were taken in 5 replicates.

##### Soluble Protein Content

Total soluble proteins were determined using the Bradford method [27] in a 96-well plate format, as reported by Dziurka et al. [34]. The absorbance was read at 595 nm and the measurements were taken in 5 replicates.

##### Total Extractable Antioxidants

Total extractable antioxidants were measured using the CUPRAC method [41]. Both hydrophilic and lipophilic fractions (50 µL each) were analyzed as described by Dziurka et al. [42], using a Cu^2+^ neocuproine complex in a 1 M ammonia–acetate buffer at pH 7.0. After 15 min of incubation, absorbance was recorded at 425 nm. The antioxidant content was calculated as Trolox-Equivalent Antioxidant Capacity (TEAC) in mmol/mg and the measurements were taken in 5 replicates.

##### Non-Enzymatic Antioxidant Capacities of Soluble Proteins

The hydrophilic fraction (protein extract) was utilized for this analysis. Following the methodology of Cekiç et al. [43], soluble proteins were precipitated using 5% *w/v* trichloroacetic acid (TCA). The samples were then centrifuged, discarding the supernatant, and the remaining pellet was prepared for further analysis. The protein fraction was redissolved in a buffer containing 50 mM Tris, 2% SDS, and 8 M urea (pH 7) before being subjected to a modified CUPRAC method. Analyses were conducted as reported by Dziurka et al. [42], substituting acetate buffer with the 50 mM Tris-8 M urea buffer. Absorbance was recorded at 425 nm, and the antioxidant content was calculated as Trolox-Equivalent Antioxidant Capacity (TEAC) in mmol/mg. The measurements were taken in 5 replicates.

##### Total Antioxidant Capacity of Insoluble and Matrix-Bound Matter

The antioxidant capacity of the insoluble and matrix-bound matter was assessed using the QUENCHER-CUPRAC method [44], conducted in a well-plate format as detailed by Dziurka et al. [42]. The remaining pellet of the insoluble fraction was combined with 10 mM Cu^2+^, 7.5 mM neoprene, and a 1 M ammonia–acetate buffer (pH 7.0), along with methanol, to achieve a final volume of 1.5 mL in a test tube. After shaking for 30 min, the samples were centrifuged, and the supernatant was transferred to 96-well plates. Absorbance was measured at 425 nm, and the antioxidant response was calculated as Trolox-Equivalent Antioxidant Capacity (TEAC) in mmol/mg. The measurements were taken in 5 replicates.

##### Hydrogen Peroxide Content

The hydrogen peroxide (H_2_O_2_) content was determined according to Ishikawa et al. [45]. Flag leaves were homogenized in an extraction buffer (1.4 mL) containing potassium phosphate buffer (50 mM, pH 7.5), trichloroacetic acid (5%), EDTA (1 mM), and polyvinylpyrrolidone (1% *w*/*v*). The reaction mixture consisted of 2.5 mL homovanillic acid (1.25 mM), 2.5 µL horseradish peroxidase (1380 U∙mg^−1^) and 20 µL leaf extract. The hydrogen peroxide content was determined using a Perkin-Elmer LS 50B spectrofluorometer (Norwalk, CT, USA). The samples were excited at 315 nm and fluorescence was detected between 400 and 450 nm. The slit widths of the excitation and emission monochromators were set at 10 nm and the measurements were taken in 5 replicates.

#### 2.3.11. Determination of Fresh/Dry Weight and Yield Components

The fresh weight of flag leaves was measured immediately after harvesting. For dry weight determination, the same leaf samples were placed in oven at 100 °C for 24 h to achieve a constant mass. After drying, samples were weighed again to obtain the dry weight. The measurements were taken in 15 replicates.

Yield-related parameters were evaluated at the maturity stage. For each treatment, 20 randomly selected plants were analyzed. The following traits were analyzed: the main shoot length, number of lateral shoots, straw biomass, length of the main shoot ear, ear weight, grain number of main/lateral shoot, grain weight of main/lateral shoot, weight of the de-grained ear, total number of grains, total grain weight, and thousand-grain weight. The measurements were taken in 20 replicates.

### 2.4. Statistical Analyses

All statistical analyses were performed using STATISTICA software, version 13.0 (StatSoft, Inc., Tulsa, OK, USA). The results are presented as means accompanied by standard error (SE). Differences between treatment groups were assessed using Student’s *t*-test. Asterisks indicate statistically significant differences at a threshold of *p* ≤ 0.05.

## 3. Results

### 3.1. Soluble Phenolics, PAL and TAL Activities, Carbohydrates, and the Water Status of the Flag Leaves

*4*-hydroxybenzoic hydrazide (HBH) was detected in the flag leaf after treatment with 10^−3^ M HBH, while it was not detected in the control (Table 1). In addition to this, there was a significant decrease in the content of soluble phenolic compounds, which dropped from 15.9 [µg/mg (DW)] in the control to 8.7 [µg/mg (DW)] (reduced by 45%) following HBH application. The *L*-phenylalanine ammonia-lyase (PAL) activity slightly decreased in the presence of the inhibitor compared to the control, although the difference was not statistically significant. Conversely, the treatment led to a marked increase in *L*-tyrosine ammonia-lyase (TAL) activity (133% increase) (Table 1).

**Figure 1:**
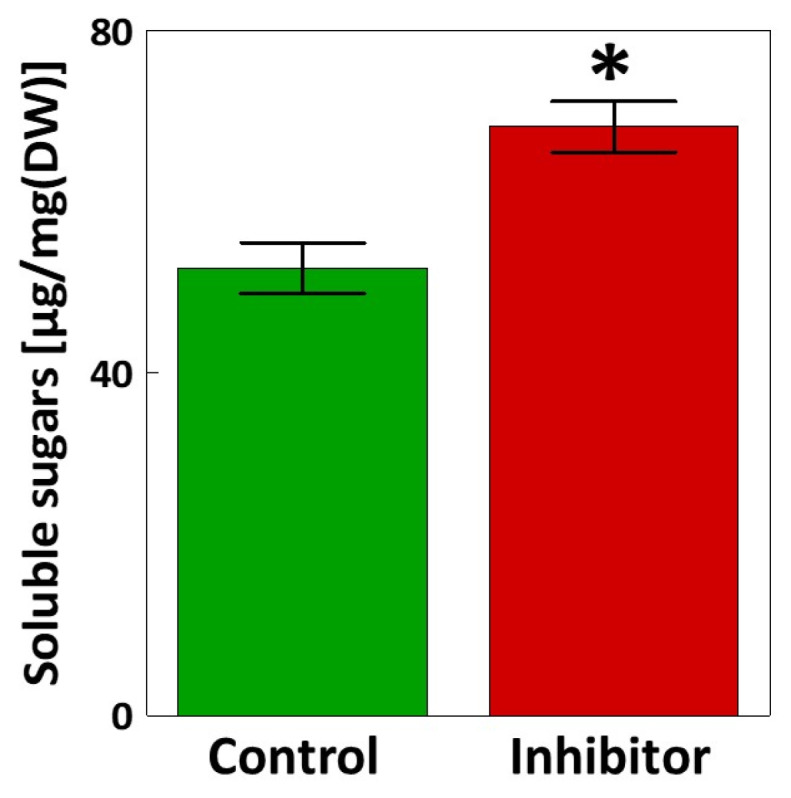
Soluble carbohydrates content [µg/mg(DW)] in the flag leaves treated with the PAL inhibitor. Bars represent mean values ± SE (n = 7). An asterisk indicates a statistically significant difference between the inhibitor and control determined by Student’s *t*-test (*p* < 0.05).

The decrease in the content of soluble phenolic compounds was accompanied by a significant increase in the content of soluble carbohydrates (32% increase) in the flag leaves of triticale under HBH-treatment (Figure 1).

**Figure 2:**
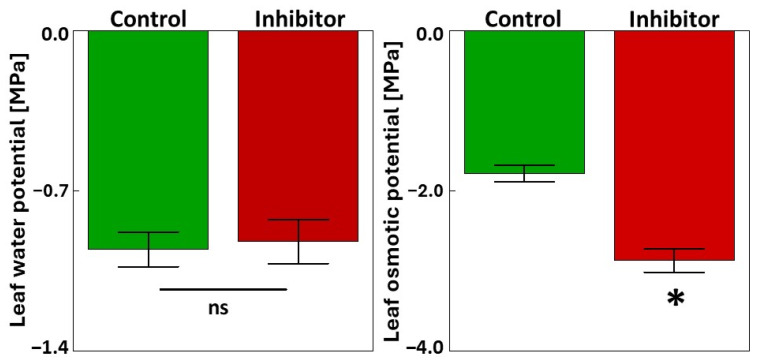
Water potential and osmotic potential in the flag leaves treated with the PAL inhibitor. Bars represent mean values ± SE (n = 7). An asterisk indicates a statistically significant difference between the inhibitor and the control determined by Student’s *t*-test (*p* < 0.05), ns indicates no statistically significant difference.

The water status of the flag leaves was also examined after treatment with the PAL inhibitor. Figure 2 shows the changes in the water potential and osmotic potential of flag leaves. No significant changes were observed in the water potential between the control and HBH-treated plants. However, a significant increase in osmotic potential was detected in the flag leaves treated with 10^−3^ M HBH compared to the control (61% increase).

### 3.2. Photosynthesis, Chlorophyll Fluorescence, Chlorophyll Content, and Accumulation of Selected Proteins

A significant decrease in the net photosynthesis rate was observed in the flag leaves of triticale (from 20.84 to 17.82 µmol/m^2^/s; 14.5% decrease) under HBH treatment (Table 2). Stomatal conductance also significantly decreased (from 639.8 to 496.4 mmol/m^2^/s; 22% decrease), while the transpiration rate and intercellular CO_2_ concentration remained unchanged. No significant differences were found in the stomatal limitation value or intrinsic water use efficiency. However, measurements revealed a significant decrease in both the apparent carboxylation efficiency (from 0.063 to 0.054 mol/m^2^/s; 14% decrease) and instantaneous water use efficiency (from 3.16 to 2.80 µmol/mmol; 11% decrease) after treatment with the PAL inhibitor (Table 2).

Statistically significant increases in the quantum yield of PSII (from 0.844 to 0.859), the quantum efficiency of PSII (from 0.282 to 0.328) and photochemical quenching coefficient (from 0.515 to 0.584) were recorded in the flag leaves of triticale under HBH treatment (Table 3). No significant differences were observed in the values of maximum efficiency of PSII (F_v_’/F_m_’), non-photochemical quenching (q_N_), electron transport rate (ETR) or chlorophyll level (Chl).

**Figure 3:**
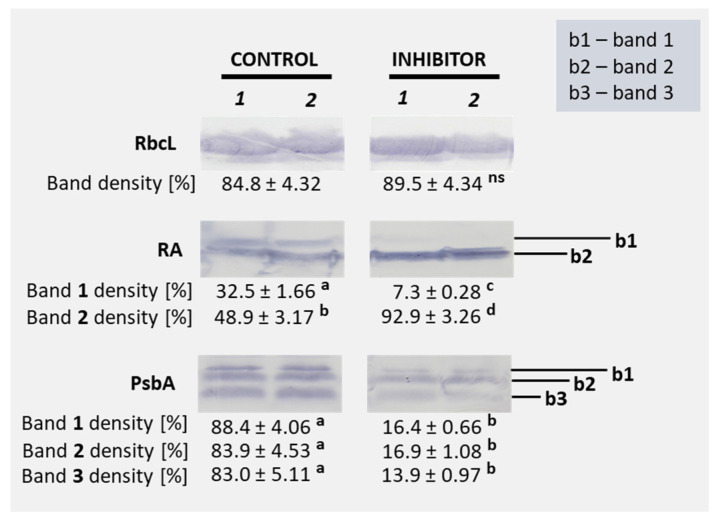
Accumulation of selected proteins (RbcL—the large subunit of Rubisco, RA—Rubisco activase, PsbA—D_1_ protein of PSII) in the flag leaves of triticale plants treated with 10^−3^ M HBH. Mean values ± SE (n = 4) for densitometric quantifications (%). Values marked with the same letter for individual bands within RA and PsbA are not significantly different, as determined by Duncan’s multiple range test (*p* < 0.05). For RbcL, differences in band density between control and inhibitor-treated samples were evaluated using Student’s *t*-test (*p* < 0.05), ns indicates no statistically significant difference.

Figure 3 shows the accumulation of selected proteins—the large subunit of Rubisco (RbcL), Rubisco activase (RA), and PsbA (D_1_ protein of PSII)—in the flag leaves of triticale under control and HBH treatment conditions. Protein accumulation is shown as band density [%]; an increase in band density reflects higher protein accumulation in the flag leaves. No statistically significant differences were observed in the accumulation of RbcL between control and inhibitor-treated samples (84.8% vs. 89.5%). In the case of Rubisco activase (RA), a significant reduction in band 1 density was detected under HBH treatment (7.3%) compared to the control (32.5%), while band 2 density significantly increased (92.9%) relative to the control (48.9%). For PsbA, HBH treatment led to a marked decrease in the density of all three detected bands, with the band 1 density dropping from 88.4% in the control to 16.4% in the treated samples, band 2 density from 83.9% to 16.9%, and band 3 density from 83.0% to 13.9% (Figure 3 and Figure S1).

### 3.3. Non-Enzymatic and Enzymatic Antioxidants and Hydrogen Peroxide

A significant decrease in the antioxidant capacity, associated with non-enzymatic soluble proteins, was observed in the flag leaves of triticale treated with 10^−3^ M HBH (from 7.79 to 5.16 nmol/mg) (34% decrease) (Table 4). Treatment with HBH resulted in a significant increase in the antioxidant capacity of the water-soluble (H_2_O) fraction (from 45.0 to 63.1 nmol/mg) (40% increase), whereas no significant changes were detected in the methanol-soluble (MeOH) and insoluble (IF) fractions. The total antioxidant capacity, calculated as the sum of the H_2_O, MeOH, and IF fractions, was significantly higher after HBH treatment (increasing from 92.6 to 111.3 nmol/mg) (20% increase) (Table 4).

A significant increase in the activity of all analyzed antioxidant enzymes was observed following treatment with PAL compared to the control (Table 5). Superoxide dismutase (SOD) activity increased from 1361.5 to 2843.7 [U/mg(prot.)] (109% increase), while total soluble peroxidase (POX) activity rose significantly from 1067.3 to 3315.3 [µM/min./mg(prot.)] (211% increase). Similarly, catalase (CAT) activity was notably elevated, increasing from 179.5 to 360.5 [mM/min./mg(prot.)] (101% increase).

*L*-ascorbate peroxidase (AsPOX) showed a marked increase from 2286.6 to 5283.1 [µM/min./mg(prot.)] (131% increase). Glutathione peroxidase (GPx) and glutathione reductase (GR) activities also increased significantly, from 14.30 to 25.75 [µM/min./mg(prot.)] (80% increase) and from 1230.6 to 2195.9 [µM/min./mg(prot.)] (78% increase), respectively. Additionally, a significant reduction in hydrogen peroxide (H_2_O_2_) content was observed in HBH-treated plants compared to the control, with the H_2_O_2_ level decreasing from 1.21 [nmol/g(FW)] to 0.81 [nmol/g(FW)] (33% decrease) in the PAL-inhibited plants (Table 5).

### 3.4. Fresh and Dry Weight and Yield-Related Traits

**Figure 4:**
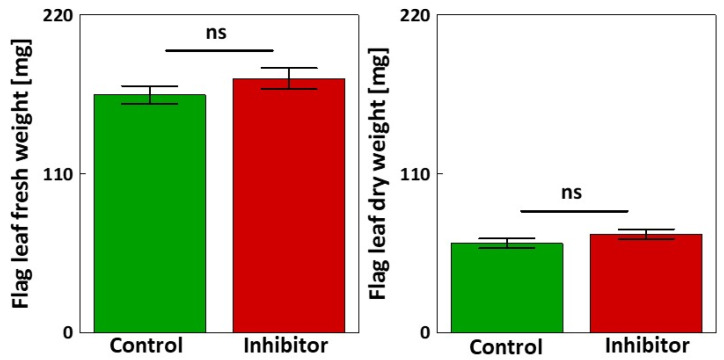
Fresh weight [mg] and dry weight [mg] of flag leaves of triticale plants treated with 10^−3^ M HBH compared to the control. Mean values ± SE (n = 15). ns indicates no statistically significant difference between treatments, as determined by Student’s *t*-test (*p* > 0.05).

No statistically significant differences were observed in either the fresh or dry weights of flag leaves between the control and plants treated with 10^−3^ M HBH (Figure 4).

Similarly, no statistically significant differences were observed between the control and inhibitor-treated plants for most yield-related traits. However, a significant difference was noted in the length of the main shoot ear (decrease of 9%) and in the weight of the de-grained ear of the main shoot (decrease of 18%) following treatment with 10^−3^ M HBH (Table 6).

## 4. Discussion

Inhibition of PAL in the flag leaves of triticale confirmed the key role of this enzyme in the synthesis of phenolic compounds [46], as evidenced by the observed decreases in their contents (Table 1). The increase in TAL activity suggests a compensatory response that maintains the phenolic level through an alternative pathway [47]. The compensatory role of other enzymes within the phenylpropanoid pathway in response to the limited PAL activity has been discussed in other studies indicating metabolic flexibility [48,49,50]. It should be emphasized that in monocots, certain PAL isoforms are bifunctional, as they can utilize both phenylalanine and tyrosine as substrates. Hence, these isoforms also display TAL activity, enabling the incorporation of *L*-tyrosine into the phenylpropanoid pathway via *p*-coumarate formation [51,52].

Application of HBH resulted in a significant increase in soluble carbohydrate levels in flag leaves (Figure 1). The relationship between carbohydrate and phenolic metabolism has previously been described in several studies [20,53,54,55]. It is well established that an increase in soluble carbohydrates, i.e., glucose and sucrose, can stimulate the production of phenolic compounds, as carbohydrates serve as carbon sources for the phenylpropanoid pathway. Grace and Logan [56] showed that elevated levels of soluble sugars can enhance phenolic compound synthesis as part of the plant’s defense mechanism under high light conditions, low temperatures, pathogen infection and nutrient deficiency. Glycosylation is another important link between carbohydrates and phenolic metabolism, increasing the solubility, transport, and biological activity of phenolic compounds in plants [57,58]. For example, Tognetti et al. [59] demonstrated that soluble carbohydrates were used in the glycosylation of auxin indole-*3*-butyric acid (IBA) by UDP-glucosyltransferase UGT74E2 under water stress conditions, which improved stress tolerance. By analogy, the reduced PAL activity in the flag leaves of triticale may limit phenolic glycosylation, leading to carbohydrate accumulation and their possible redirection toward osmotic adjustment [60], energy storage [61] or the glycosylation of other compounds, such as auxins [62], abscisic acid [63], cytokinins [64], and brassinosteroids [65]. These observations uncover a new perspective of how carbon is partitioned between primary (sugar-related) and secondary (phenolic-related) metabolism in plants under PAL inhibition.

The observed decrease in the net photosynthesis rate and stomatal conductance, with no significant change in intercellular CO_2_ concentration (Table 2), suggests that the limitation of photosynthesis under PAL inhibition involves both stomatal and non-stomatal (biochemical) factors. This is further confirmed by the reduction in apparent carboxylation efficiency (A_m_ = P_N_/C*_i_*), which indicates how effectively CO_2_ is converted into carbohydrates in the mesophyll [29]. The lower A_m_ values observed in HBH-treated plants indicate the decreased carboxylation efficiency of Rubisco (Table 2).

These results are consistent with the changes in Rubisco activase isoforms observed under PAL inhibition (Figure 3), suggesting impaired regulation of Rubisco, a key enzyme controlling carboxylation efficiency in plants [66]. Rubisco activase (RA) occurs in two isoforms, a larger isoform (*α*, 46 kDa) and a smaller isoform (*β*, 43 kDa), which are produced via alternative splicing or separate gene expression [67]. These isoforms are differentially regulated at the post-transcriptional level [68] and their expression is affected by both environmental and metabolic conditions [69]. The larger isoform contains two cysteine residues that allow for redox regulation by thioredoxin, enabling it to respond to oxidative conditions. In contrast, the smaller isoform lacks this redox-sensitive region and is primarily regulated by the ATP/ADP ratio [70]. Under control conditions, both isoforms were detected in the flag leaves of triticale. However, HBH treatment strongly reduced the accumulation of the larger isoform (b1), while promoting increased levels of the smaller isoform (b2) (Figure 3). This result reflects impaired redox regulation of Rubisco activase under PAL inhibition, leading to preferential accumulation of the smaller isoform, which, unlike the redox-regulated larger isoform, primarily responds to changes in the ATP/ADP ratio caused by light-dependent reactions. Given that PAL inhibition alters phenolic metabolism and redox balance, the reduced abundance of the larger isoform may reflect impaired redox signaling. At the same time, the upregulation of the smaller isoform may reflect a compensatory response to maintain Rubisco activation. This suggestion is consistent with the observed changes in carboxylation efficiency and photosynthetic capacity in HBH-treated plants (Table 2, Figure 3).

Despite the decline in net photosynthesis, the enhanced PSII quantum yield (F_v_/F_m_), PSII quantum efficiency (Φ_PSII_) and photochemical quenching of PSII (q_P_) suggest a photoprotective adjustment of the photosynthetic apparatus under PAL inhibition (Table 3). A similar response was reported by Jia and Lu [71], who found that treatment of maize plants with abscisic acid (ABA) under strong light conditions led to an increased PSII quantum yield (F_v_/F_m_), PSII quantum efficiency (Φ_PSII_), and photochemical quenching (q_P_). Therefore, our results are a valuable confirmation that plants may upregulate photochemical processes to mitigate photoinhibition and maintain photosynthetic electron transport efficiency [72]. However, a significant decrease in PsbA (D_1_ protein) accumulation was observed under HBH treatment (Figure 3). This suggests that the effect of HBH on the D_1_ degradation process is stronger than that on D_1_ synthesis itself [73]. Moreover, the decrease in phenolic compounds, which function as ROS scavengers [74] and photoprotectors [75], may further affect PSII function. These results suggest that PAL inhibition affects not only carbon allocation, but also the activity and stability of the photosynthetic apparatus.

The marked increase in the activity of antioxidant enzymes (SOD, CAT, POX, AsPOX, GPx, and GR) (Table 5), alongside enhanced total antioxidant capacity (Table 4), suggests that the decrease in phenolic content induced by PAL inhibition greatly influenced enzymatic antioxidant activation. This reveals the close relationship between non-enzymatic (phenolic-based) and enzymatic antioxidant defenses, reflecting their functional complementarity in maintaining redox homeostasis [76]. Enzymatic upregulation was accompanied by a significant decrease in hydrogen peroxide (H_2_O_2_) levels, suggesting that the antioxidant defense system effectively mitigated ROS accumulation. Although the antioxidant role of phenolic compounds is well documented, there is a notable lack of studies investigating how their depletion influences the activity of enzymatic antioxidants. Analyses of non-enzymatic and enzymatic antioxidants have mainly been conducted in the context of environmental stresses [77,78] to assess oxidative stress levels in plants [79,80], and the interrelationship between these two antioxidant systems has not been explored. Our findings demonstrate a compensatory upregulation of enzymatic antioxidants in response to reduced phenolic levels, addressing a critical gap in the current knowledge. This provides novel evidence of the dynamic interplay between enzymatic and non-enzymatic antioxidant pathways and highlights the multifaceted role of phenolics beyond their direct ROS-scavenging capacity.

While biomass and most yield components remained unaffected (Figure 4), the significant reductions in ear length and the weight of the de-grained ear suggest that reproductive development may be more sensitive to phenolic metabolism. As highlighted by Barros Santos et al. [81] and Wagay et al. [82], phenolics are involved in a range of physiological processes, including reproductive organ development. Our findings extend this knowledge by implicating PAL activity in triticale spike development under optimal growth conditions. Furthermore, although photosynthetic activity was enhanced following PAL inhibition (Table 2), this did not result in an increased grain yield (Table 6). This discrepancy indicates that other factors, such as assimilate allocation [83], may limit yield improvements. It is also possible that carbohydrates are utilized in other metabolic pathways [84] or for the maintenance of antioxidant systems [85]. Moreover, the timescale of the experiment may not be sufficient to capture long-term effects on yield formation.

## 5. Conclusions

Inhibition of PAL activity in triticale leads to major metabolic and physiological changes, including reduced phenolic contents, altered photosynthetic performance, and acute activation of antioxidant systems. The marked increase in both enzymatic and non-enzymatic antioxidant activity suggests a compensatory response aimed at maintaining redox homeostasis in conditions of limited phenolic availability. This highlights the functional link between different antioxidant systems and underlines the importance of phenolics not only as antioxidants but also as regulators of other physiological processes.

Changes in the relative abundance of Rubisco activase isoforms indicate that disturbances in phenylpropanoid metabolism may also influence photosynthesis regulation. This suggests an interaction between secondary metabolism and photosynthetic processes.

Understanding the role of PAL and phenolic metabolism in these processes has important implications for improving crop productivity. By clarifying how phenolic compounds affect photosynthetic efficiency and metabolic regulation, this knowledge can contribute to strategies aimed at optimizing yield potential in triticale and other cereals under various growing conditions. Furthermore, PAL inhibition was shown to affect ear length and grainless ear weight, which are important components influencing the overall grain yield. These findings enhance our understanding of how phenolic metabolism impacts not only physiological processes but also morphological traits directly related to productivity.

Future studies should aim to explore the genetic mechanisms underlying these responses, including the regulation of antioxidant and photosynthetic pathways under PAL inhibition. Additionally, long-term experiments using multiple genotypes are required to evaluate the potential impacts on yield and to assess the variability in responses to phenolic metabolism disruption.

## Figures and Tables

**Table 1:** Content of *4*-hydroxybenzoic hydrazide (HBH) [µg/g(DW)], soluble phenolic compounds [µg/mg(DW)], and the activity of PAL [nmol(cinnamic acid)/h/mg(prot.)] and TAL [nmol(*p*-coumaric acid)/h/mg(prot.)] enzymes in the flag leaves of triticale treated with 10^−3^ M HBH. Mean values ± SE (n = 7).

Measurements	Control	Inhibitor [10^−3^ M]
*4*-hydroxybenzoic hydrazide (HBH)	0.00 ± 0.00	4.81 ± 0.44 *
Soluble phenolics	15.9 ± 0.81	8.7 ± 0.91 *
*L*-phenylalanine ammonia-lyase (PAL)	2.42 ± 0.21	2.32 ± 0.22
*L*-tyrosine ammonia-lyase (TAL)	0.235 ± 0.038	0.548 ± 0.074 *

Asterisks indicate statistically significant differences between the inhibitor and the control determined by Student’s *t*-test (*p* < 0.05).

**Table 2:** Net photosynthesis rate [µmol/m^2^/s], transpiration rate [mmol/m^2^/s], stomatal conductance [µmol/m^2^/s], intercellular concentration of CO_2_ [µmol/mol], stomatal limitation value, apparent carboxylation efficiency [mol/m^2^/s], intrinsic water use efficiency [µmol/µmol], and instantaneous water use efficiency [µmol/mmol] in the flag leaves of triticale plants treated with 10^−3^ M HBH. Mean values ± SE (n = 10).

Measurements	Control	Inhibitor [10^−3^ M]
Net photosynthesis rate—P_N_	20.84 ± 0.48	17.82 ± 0.46 *
Transpiration rate—E	6.60 ± 0.06	6.39 ± 0.19
Stomatal conductance—*g*_S_	639.8 ± 31.4	496.4 ± 55.2 *
Intercellular concentration of CO_2_—C*_i_*	329.6 ± 2.4	328.3 ± 3.1
Stomatal limitation value—L_S_	0.161 ± 0.008	0.177 ± 0.012
Apparent carboxylation efficiency—P_N_/C*_i_*	0.063 ± 0.002	0.054 ± 0.002 *
Intrinsic water use efficiency—WUE_intr._	0.033 ± 0.002	0.039 ± 0.003
Instantaneous water use efficiency—WUE_inst._	3.16 ± 0.09	2.80 ± 0.08 *

An asterisk indicates a statistically significant difference between the inhibitor and control, determined by Student’s *t*-test (*p* < 0.05).

**Table 3:** Chlorophyll fluorescence parameters and chlorophyll content [a.u.] in the flag leaves of triticale plants treated with 10^−3^ M HBH. Mean values ± SE (n = 7).

Measurements	Control	Inhibitor [10^−3^ M]
Quantum yield of PSII—F_v_/F_m_	0.844 ± 0.006	0.859 ± 0.001 *
Maximum efficiency of PSII—F_v_’/F_m_’	0.546 ± 0.008	0.561 ± 0.008
PSII quantum efficiency—Φ_PSII_	0.282 ± 0.012	0.328 ± 0.011 *
Photochemical quenching coefficient—q_P_	0.515 ± 0.017	0.584 ± 0.012 *
Non-photochemical quenching—q_N_	0.806 ± 0.006	0.818 ± 0.007
Electron transport rate—ETR	1.48 ± 0.19	1.81 ± 0.25
Chlorophyll level—Chl	22.06 ± 0.97	22.39 ± 1.48

An asterisk indicates a statistically significant difference between the inhibitor and control, determined by Student’s *t*-test (*p* < 0.05).

**Table 4:** Antioxidant capacities [nmol/mg] of non-enzymatic soluble proteins, the water-soluble (H_2_O) fraction, the methanol-soluble (MeOH) fraction, and the insoluble fraction (IF), and the total antioxidant capacity (H_2_O + MeOH + IF) of extracts from the flag leaves of triticale treated with 10^−3^ M HBH. Mean values ± SE (n = 5).

Antioxidant Capacities	Control	Inhibitor [10^−3^ M]
Non-enzymatic soluble proteins	7.79 ± 0.79	5.16 ± 0.31 *
H_2_O fraction	45.0 ± 3.84	63.1 ± 1.28 *
MeOH fraction	28.8 ± 1.06	30.1 ± 1.13
Insoluble fraction (IF)	18.8 ± 0.36	18.0 ± 0.37
H_2_O + MeOH + IF	92. 6 ± 4.42	111.3 ± 2.28 *

An asterisk indicates a statistically significant difference between the inhibitor and control, as determined by Student’s *t*-test (*p* < 0.05).

**Table 5:** Activity of total superoxide dismutase (SOD) [U/mg(prot.)], total soluble peroxidase (POX) [µM/min./mg(prot.)], total catalase (CAT) [µM/min./mg(prot.)], *L*-ascorbate peroxidase (AsPOX) [µM/min./mg(prot.)], glutathione peroxidase (GPx) [µM/min./mg(prot.)], and glutathione reductase (GR) [µM/min./mg(prot.)], and hydrogen peroxide content (H_2_O_2_) [nmol/g(FW)] in the flag leaves of triticale treated with 10^−3^ M HBH. Mean values ± SE (n = 5).

Measurements	Control	Inhibitor [10^−3^ M]
Superoxide dismutase—SOD	1361.5 ± 149.9	2843.7 ± 222.8 *
Total soluble peroxidase—POX	1067.3 ± 67.9	3315.3 ± 387.3 *
Catalase—CAT	179.5 ± 18.9	360.5 ± 33.6 *
*L*-Ascorbate peroxidase—AsPOX	2286.6 ± 287.5	5283.1 ± 706.3 *
Glutathione peroxidase—GPx	14.30 ± 1.60	25.75 ± 1.62 *
Glutathione reductase—GR	1230.6 ± 85.5	2195.9 ± 101.0 *
Hydrogen peroxide—H_2_O_2_	1.21 ± 0.11	0.81 ± 0.13 *

An asterisk indicates a statistically significant difference between the inhibitor and control, as determined by Student’s *t*-test (*p* < 0.05).

**Table 6:** Yield-related traits of triticale plants treated with 10^−3^ M HBH. Mean values ± SE (n = 20).

Measurements	Control	Inhibitor [10^−3^ M]
Main shoot length [cm]	77.5 ± 1.86	77.7 ± 2.40
Number of lateral shoots/per plant	0.56 ± 0.11	0.70 ± 0.15
Straw biomass [g]	3.96 ± 0.21	3.51 ± 0.24
Length of the main shoot ear [cm]	9.42 ± 0.15	8.58 ± 0.19 *
Ear weight—main shoot [g]	1.99 ± 0.09	1.83 ± 0.13
Grain number—main shoot ear	36.50 ± 2.64	35.30 ± 2.39
Grain weight from the ear—main shoot [g]	1.51 ± 0.09	1.42 ± 0.09
Weight of the de-grained ear—main shoot [g]	0.481 ± 0.011	0.396 ± 0.016 *
Number of grains—lateral shoots	26.6 ± 6.52	27.0 ± 6.53
Grain weight—lateral shoots [g]	0.79 ± 0.22	0.82 ± 0.21
Total number of grains	81.2 ± 4.01	75.1 ± 4.28
Total grain weight [g]	2.87 ± 0.14	2.60 ± 0.20
Thousand-grain weight [g]	35.7 ± 1.12	34.6 ± 1.66

An asterisk indicates a statistically significant difference between the inhibitor and control, as determined by Student’s *t*-test (*p* < 0.05).

## Data Availability

All data are contained within the article. The datasets used and analyzed during the current study are available from the corresponding author on reasonable request.

## Supplementary Materials

The following supporting information can be downloaded at: https://www.mdpi.com/article/10.3390/cells14171368/s1, Figure S1. Representative original western blot membranes showing thea ccumulation of selected proteins in flag leaf tissues of triticale plants treated with 10^−3^ M *4*-hydroxybenzoic hydrazide (HBH). Detected proteins include RbcL (large subunit of Rubisco), RA (Rubiscoactivase), and PsbA (D1proteinofphotosystemII). C–control, I–inhibitor.
