# Supplementary material for: Physiological and Molecular Responses of the Flag Leaf Under L-Phenylalanine Ammonia-Lyase Inhibition
Source: Cells. 2025 Sep 2;14(17):1368. doi: 10.3390/cells14171368 (PMC12428189; doi:10.3390/cells14171368)
Supplement: Supplementary file 1 [file cells-14-01368-s001.zip › cells-3808588-supplementary.pdf]

Figure S1. Representative original western blot membranes showing the accumulation of selected proteins in flag leaf tissues of triticale plants treated with 10^{-3} M *4*-hydroxybenzoic hydrazide (HBH). Detected proteins include RbcL (large subunit of Rubisco), RA (Rubisco activase), and PsbA (D1 protein of photosystem II). C – control, I – inhibitor.

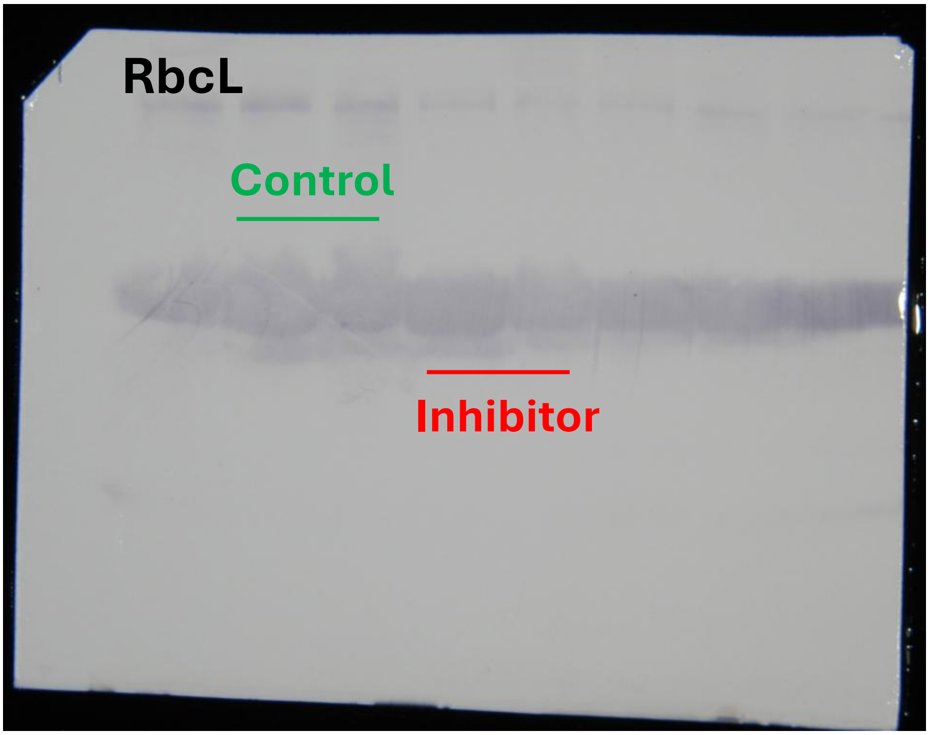

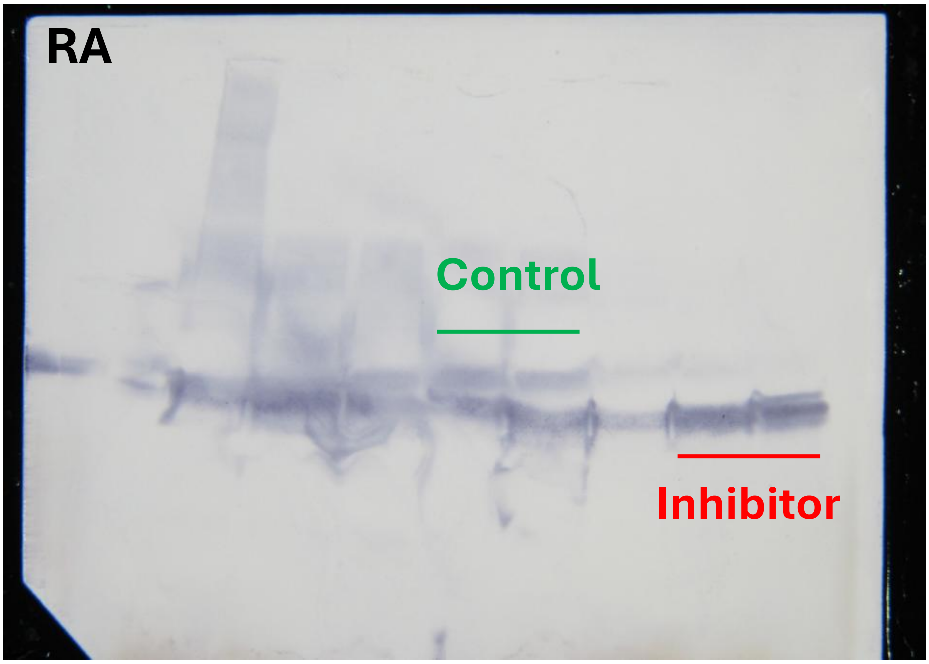

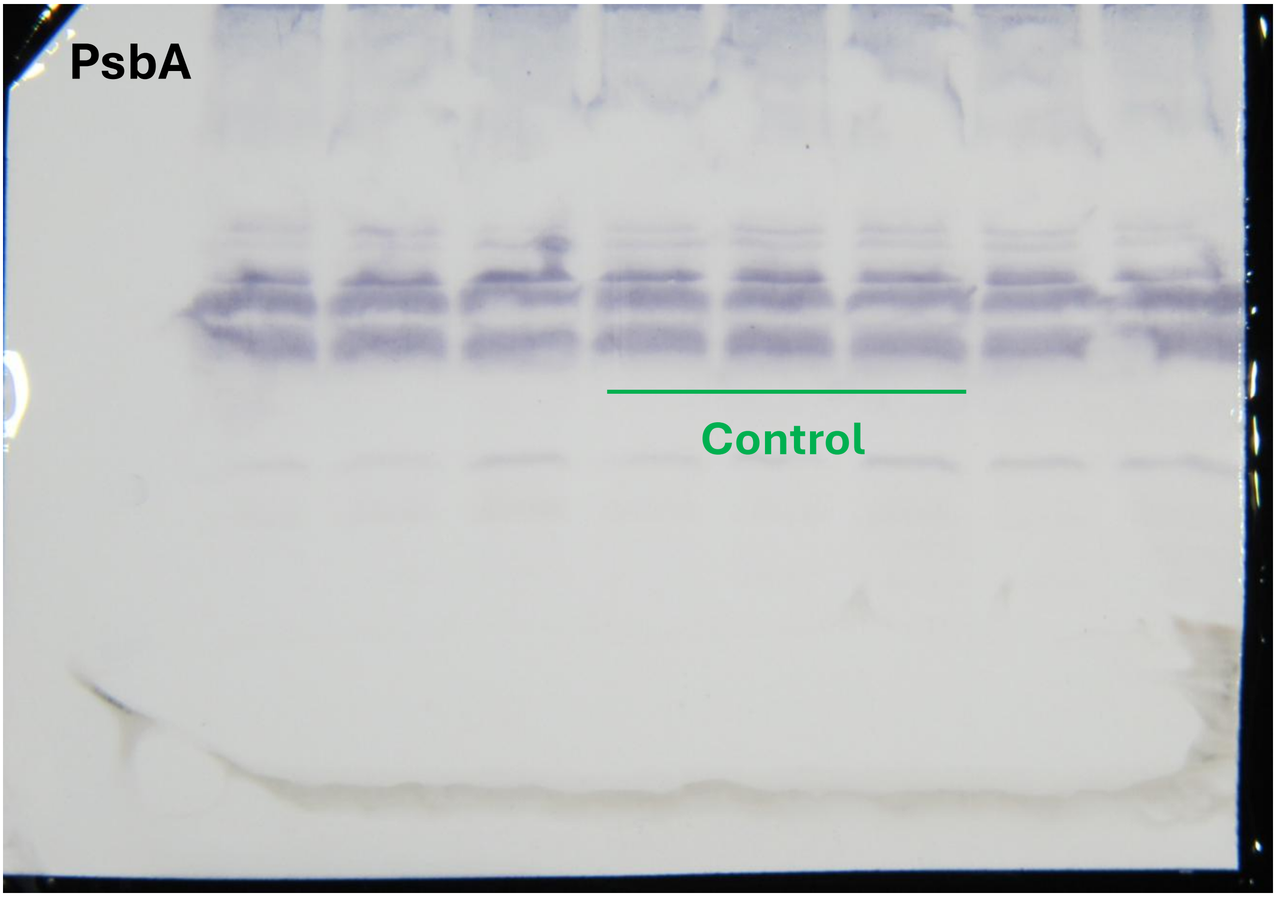
PsbA
Control

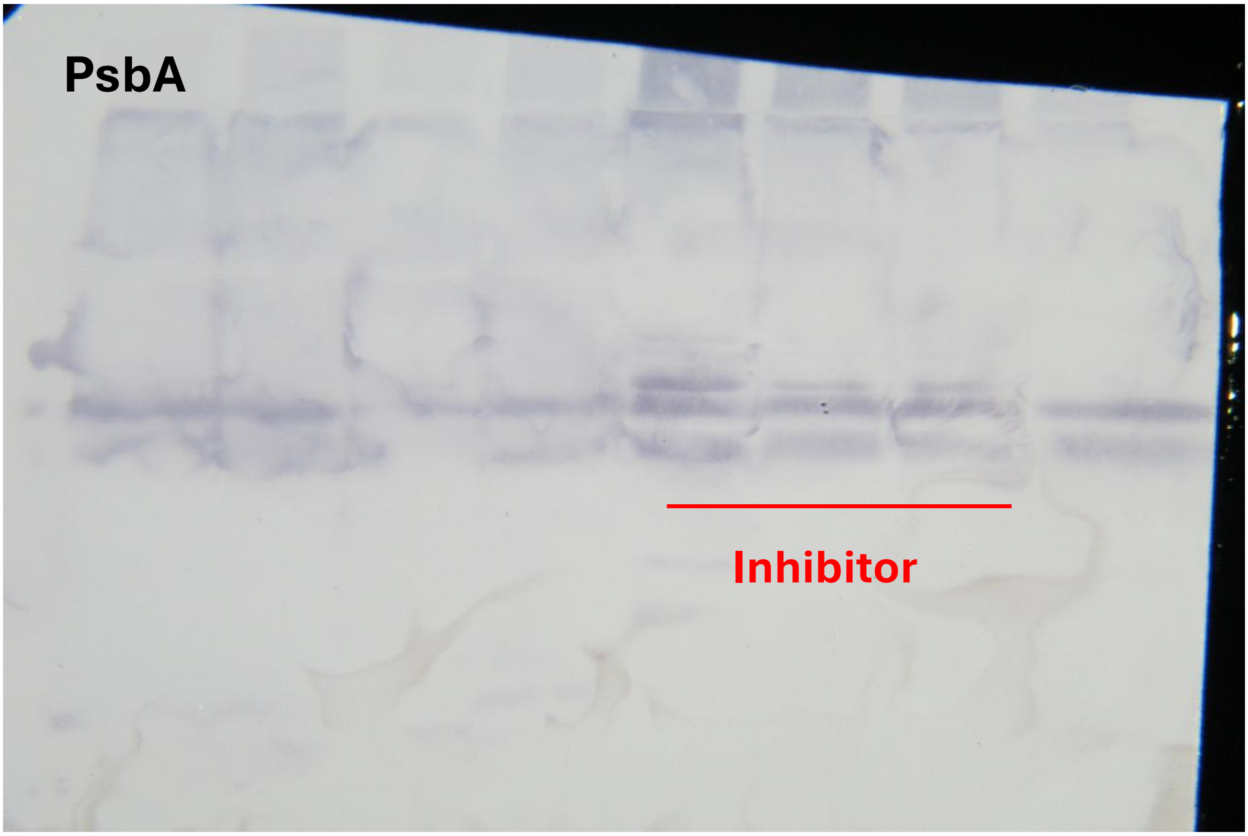
PsbA
Inhibitor
